# Efficient ammonia synthesis over a Ru/La_0.5_Ce_0.5_O_1.75_ catalyst pre-reduced at high temperature†
†Electronic supplementary information (ESI) available: Detailed procedures for each method, catalytic performance, STEM-EDX images, and detailed characterizations. See DOI: 10.1039/c7sc05343f


**DOI:** 10.1039/c7sc05343f

**Published:** 2018-01-15

**Authors:** Yuta Ogura, Katsutoshi Sato, Shin-ichiro Miyahara, Yukiko Kawano, Takaaki Toriyama, Tomokazu Yamamoto, Syo Matsumura, Saburo Hosokawa, Katsutoshi Nagaoka

**Affiliations:** a Department of Integrated Science and Technology , Faculty of Science and Technology , Oita University , 700 Dannoharu , Oita 870-1192 , Japan . Email: nagaoka@oita-u.ac.jp; b Elements Strategy Initiative for Catalysts and Batteries , Kyoto University , 1-30 Goryo-Ohara, Nishikyo-ku , Kyoto 615-8245 , Japan; c The Ultramicroscopy Research Center , Kyushu University , Motooka 744, Nishi-ku , Fukuoka 819-0395 , Japan; d Department of Applied Quantum Physics and Nuclear Engineering , Kyushu University , Motooka 744, Nishi-ku , Fukuoka 819-0395 , Japan

## Abstract

Ammonia is an important feedstock for producing fertiliser and is also a potential energy carrier. However, the process currently used for ammonia synthesis, the Haber–Bosch process, consumes a huge amount of energy; therefore the development of new catalysts for synthesising ammonia at a high rate under mild conditions (low temperature and low pressure) is necessary. Here, we show that Ru/La_0.5_Ce_0.5_O_1.75_ pre-reduced at an unusually high temperature (650 °C) catalysed ammonia synthesis at extremely high rates under mild conditions; specifically, at a reaction temperature of 350 °C, the rates were 13.4, 31.3, and 44.4 mmol g^–1^ h^–1^ at 0.1, 1.0, and 3.0 MPa, respectively. Kinetic analysis revealed that this catalyst is free of hydrogen poisoning under the conditions tested. Electron energy loss spectroscopy combined with O_2_ absorption capacity measurements revealed that the reduced catalyst consisted of fine Ru particles (mean diameter < 2.0 nm) that were partially covered with partially reduced La_0.5_Ce_0.5_O_1.75_ and were dispersed on a thermostable support. Furthermore, Fourier transform infrared spectra measured after N_2_ addition to the catalyst revealed that N_2_ adsorption on Ru atoms that interacted directly with the reduced La_0.5_Ce_0.5_O_1.75_ weakened the N≡N bond and thus promoted its cleavage, which is the rate-determining step for ammonia synthesis. Our results indicate that high-temperature pre-reduction of this catalyst, which consists of Ru supported on a thermostable composite oxide with a cubic fluorite structure and containing reducible cerium, resulted in the formation of many sites that were highly active for N_2_ reduction by hydrogen.

## Abstract 2

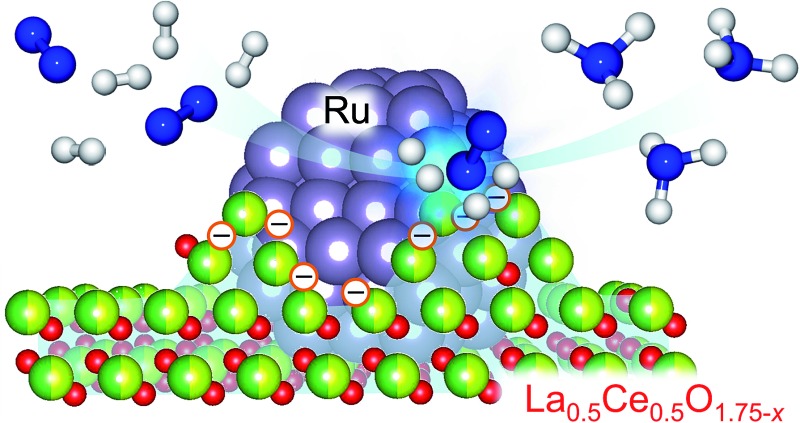
A Ru/La_0.5_Ce_0.5_O_1.75_ catalyst pre-reduced at an unusually high temperature (650 °C) catalyses ammonia synthesis at a high rate under mild conditions.

## Introduction

Ammonia is an important chemical feedstock, and more than 80% of the ammonia that is synthesised is used to produce fertiliser.^1^ Ammonia also has potential utility as an energy carrier and a hydrogen source^2^–^5^ (1) because it has a high energy density (12.8 GJ m^–3^) and a high hydrogen content (17.6 wt%), (2) because infrastructure for ammonia storage and transportation already exists, and (3) because carbon dioxide is not emitted when ammonia is decomposed to produce hydrogen.^2^,^4^,^6^,^7^ If ammonia could be efficiently produced from a renewable energy source, such as solar or wind energy, problems related to the global energy crisis could be mitigated.

Ammonia is usually synthesised by the energy-intensive Haber–Bosch process, which is performed at very high temperatures (>450 °C) and high pressures (>20 MPa) and which accounts for 1–2% of global energy consumption. Approximately 60% of the energy consumed by the process is recovered and stored as enthalpy in the ammonia molecule; but the remaining energy is lost, mostly during hydrogen production from natural gas, ammonia synthesis, and gas separation. The development of methods for reduction of the energy used by this process has been the goal of a considerable amount of research.^8^ One way to accomplish this would be to replace the iron-based catalysts used in the Haber–Bosch process with a catalyst that would permit the use of milder conditions (lower temperatures and pressures).^9^–^12^

Ammonia has been synthesised under ambient conditions with organometallic catalysts, but strong reducing agents and proton sources are generally needed, and the ammonia production rate is too low for practical applications.^13^–^15^ Supported ruthenium catalysts are good candidates for ammonia synthesis because they are more active at low temperature and pressure than iron-based catalysts are. The rate-determining step in ammonia synthesis is generally cleavage of the high-energy N≡N bond of N_2_ (945 kJ mol^–1^).^13^,^16^ One effective way to accelerate this step is to modify the Ru electronic states.^17^,^18^ This can be accomplished by the use of basic catalyst supports and by the addition of a strongly basic promoter; these modifications have been shown to enhance ammonia synthesis activity^17^,^18^ by means of a mechanism that involves the transfer of electrons to the Ru metal from the basic components and subsequent transfer of electrons from Ru to the antibonding π-orbitals of N_2_, which weakens the N≡N bond and promotes its cleavage.^19^ The most effective promoter has been reported to be Cs_2_O.^19^ The combination of Cs^+^, Ru, and MgO possesses high ammonia-synthesis activity^19^,^20^ and has been used as a benchmark in many studies.^9^,^21^ BaO is also an effective promoter, and the combination of Ba^2+^, Ru, and activated carbon has been used in industrial-scale commercial processes.^22^ Notably, Ru catalysts supported on non-oxides, such as Ru-loaded electride [Ca_24_Al_28_O_64_]^4+^(e^–^)_4_ (Ru/C12A7:e^–^) and Ru/Ca(NH_2_)_2_, also show high ammonia-synthesis activity.^9^,^23^,^24^ In fact, the ammonia-synthesis activity of Ru/Ca(NH_2_)_2_ is higher than the activities of any previously reported Ru catalysts, as well as the activities of 3d transition metal–LiH composites, which are a new class of non-Ru ammonia-synthesis catalysts.^25^ The high activities of catalysts supported on non-oxides have been attributed to the strong electron-donating ability of the supports. However, the practical utility of these catalysts might be limited by the sophisticated procedures required to prepare them and by their air and moisture sensitivity.

In the 1990s, Aika *et al.* found that rare earth oxides, such as CeO_2_ and La_2_O_3_, are effective supports for Ru catalysts.^26^ In addition, we recently reported that a Ru catalyst supported on the rare earth oxide Pr_2_O_3_ exhibits high ammonia-synthesis activity.^27^ Aika *et al.* reported that the rate of ammonia synthesis over Ru/CeO_2_ is high when the catalyst has been pre-reduced at 500 °C.^26^ During pre-reduction, some of the Ce^4+^ is reduced to Ce^3+^, and thus an electron is transferred to Ru and then to adsorbed N_2_ molecules. However, the ammonia synthesis rate is slower over a catalyst that has been pre-reduced at a temperature higher than 500 °C, owing to structural changes associated with sintering of the support. To increase the specific surface area of the catalysts, as well as the reducibility of the Ce^4+^, various investigators have used composite-oxide supports, such as CeO_2_–La_2_O_3_,^28^ MgO–CeO_2_,^29^,^30^ BaO–CeO_2_,^31^ CeO_2_–ZrO_2_,^32^ and Sm_2_O_3_–CeO_2_,^33^ for Ru catalysts. However, the ammonia-synthesis rates achieved with these catalysts remain insufficient for practical use. As suggested by Aika *et al.*, the pre-reduction temperature for these catalysts is kept below 500 °C to minimize aggregation of the Ru particles.^26^

Herein, we report the ammonia-synthesis activity of Ru/La_0.5_Ce_0.5_O_1.75_, a catalyst consisting of Ru supported on a La_0.5_Ce_0.5_O_1.75_ solid solution, which is a composite oxide of CeO_2_ and La_2_O_3_. After pre-reduction at the unusually high temperature of 650 °C, the catalyst exhibited high ammonia-synthesis activity at reaction temperatures from 300 to 400 °C; the activity was the highest among oxide supported Ru catalysts and comparable to that of the most active Ru catalysts reported to date. The thermostable oxide support, which had an average composition of La_0.5_Ce_0.5_O_1.64_ after pre-reduction at 650 °C, consisted of fine Ru particles strongly anchored to the reduced support and had numerous active Ru sites. The dependence of the catalyst structure and state on the reduction temperature was elucidated by means of various characterisation techniques, including energy electron loss spectroscopy (EELS) and scanning transmission electron microscopy (STEM). This catalyst has the advantages of being easy to prepare and stable in the atmosphere, which makes it easy to load into a reactor.

## Results and discussion

### Ammonia-synthesis activity of Ru/La_0.5_Ce_0.5_O_1.75_

**Fig. 1:**
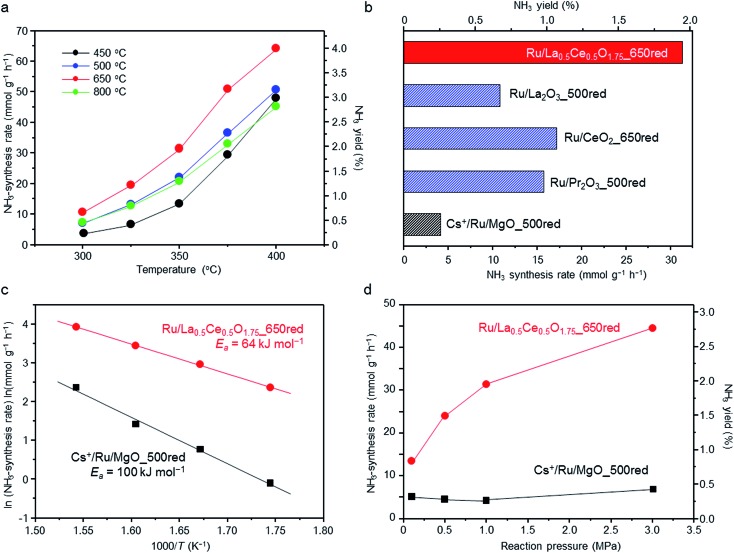
Evaluation of catalyst activities for ammonia synthesis. (a) The temperature dependence of the ammonia-synthesis rate and NH_3_ yield at 1.0 MPa over Ru/La_0.5_Ce_0.5_O_1.75_ after reduction at 450, 500, 650, or 800 °C. (b) Ammonia-synthesis rates and NH_3_ yields at 350 °C and 1.0 MPa over supported Ru catalysts, each of which had been reduced at the optimal temperature for that catalyst. (c) Arrhenius plots for ammonia-synthesis reactions at 1.0 MPa over Cs^+^/Ru/MgO_500red and Ru/La_0.5_Ce_0.5_O_1.75__650red. (d) The pressure dependence of the ammonia-synthesis rate and NH_3_ yield at 350 °C over Cs^+^/Ru/MgO_500red and Ru/La_0.5_Ce_0.5_O_1.75__650red. Reaction conditions: catalyst, 100 mg; reactant gas, 3 : 1 H_2_/N_2_ at a flow rate of 120 N mL min^–1^.

The reaction temperature dependence of the ammonia-synthesis rate over Ru/La_0.5_Ce_0.5_O_1.75_ was measured at 1.0 MPa after pre-reduction of the catalyst at 450, 500, 650, or 800 °C. Under the reaction conditions, the equilibrium ammonia-synthesis rate and the ammonia yield at 400 °C are 127 mmol g^–1^ h^–1^ and 7.91%, respectively. At all reaction temperatures, the ammonia-synthesis rate was markedly higher over the catalyst pre-reduced at 650 °C than over the catalysts pre-reduced at 450 °C (a temperature that was used in a previously reported study^28^) or 500 °C (Fig. 1a). However, increasing the pre-reduction temperature to 800 °C sharply decreased the rate. That is, the optimal pre-reduction temperature was 650 °C, which is considerably higher than the reaction temperatures usually used for Ru-catalysed ammonia synthesis (≤400 °C).

We also compared the ammonia-synthesis rates over various other supported 5 wt% Ru catalysts at 350 °C and 1.0 MPa (Fig. 1b). Each of the catalysts had been pre-reduced at a temperature between 500 and 800 °C, and the ammonia-synthesis rates after reduction at the optimal pre-reduction temperature are displayed. The ammonia-synthesis rate over Ru/La_0.5_Ce_0.5_O_1.75__650red (“650red” indicates that the catalyst had been reduced at 650 °C before the activity tests) reached 31.3 mmol g^–1^ h^–1^ and was much higher than the rates over the other tested catalysts, such as Ru/CeO_2__650red (17.2 mmol g^–1^ h^–1^) and Ru/La_2_O_3__500red (10.8 mmol g^–1^ h^–1^), whose supports each contain one of the rare earth elements in La_0.5_Ce_0.5_O_1.75_, and Ru/Pr_2_O_3__500red (15.7 mmol g^–1^ h^–1^),^27^ which is one of the most active of the oxide-supported Ru catalysts. Furthermore, the ammonia-synthesis rate over Ru/La_0.5_Ce_0.5_O_1.75__650red was approximately 7.6 times that over Cs^+^/Ru/MgO_500red (4.1 mmol g^–1^ h^–1^), a well-known catalyst that is often used as a benchmark and that is more active than Ba^2+^/Ru/activated carbon^9^,^34^ which is used commercially in ammonia-synthesis processes.^22^ Note also that the ammonia-synthesis rate over 5 wt% Ru/La_0.5_Ce_0.5_O_1.75__650red was comparable to that over 10 wt% Ru/Ca(NH_2_)_2_ (31.7 mmol g^–1^ h^–1^, measured under similar reaction conditions [340 °C, 0.9 MPa]).^24^

We prepared Arrhenius plots for ammonia-synthesis reactions catalysed by Ru/La_0.5_Ce_0.5_O_1.75__650red and Cs^+^/Ru/MgO_500red with the use of the rates at 300, 325, 350, and 375 °C (Fig. 1c). To avoid the contribution of the reverse reaction to the ammonia-synthesis rate, the rate at 400 °C was not used in the plots. The apparent activation energy (*E*_a_) calculated for Ru/La_0.5_Ce_0.5_O_1.75__650red (64 kJ mol^–1^) was much lower than that for Cs^+^/Ru/MgO_500red (100 kJ mol^–1^), and was comparable to that reported for 10 wt% Ru/Ca(NH_2_)_2_ (59 kJ mol^–1^).^24^ These results demonstrate that the low apparent activation energy for the reaction over Ru/La_0.5_Ce_0.5_O_1.75__650red was responsible for the high ammonia-synthesis rate.

We also investigated the effect of reaction pressure on ammonia-synthesis rates at 350 °C (Fig. 1d). Increasing the reaction pressure from 0.1 to 1.0 MPa reportedly has no effect on the ammonia-synthesis rate over Cs^+^/Ru/MgO_500red.^9^,^24^ This result implies that hydrogen atoms strongly adsorbed on the Ru interfere with the activation of N_2_ molecules (a phenomenon referred to as hydrogen poisoning), which is a typical drawback of conventional Ru catalysts.^35^,^36^ In contrast, we observed that at 0.1 MPa, the ammonia-synthesis rate over Ru/La_0.5_Ce_0.5_O_1.75__650red was 13.4 mmol g^–1^ h^–1^, which is the highest value reported for Ru catalysts to date; and the rate increased to 31.3 and 44.4 mmol g^–1^ h^–1^ when the pressure was increased to 1.0 and 3.0 MPa, respectively. Hence, we assumed that hydrogen poisoning did not occur over Ru/La_0.5_Ce_0.5_O_1.75__650red at the tested temperature. To confirm this assumption, we performed kinetic analysis at 350 °C and 0.1 MPa. For that purpose, reaction orders for N_2_, H_2_, and NH_3_ were determined with the assumption of the rate expression (1) (reaction conditions and obtained results are shown in Table S1†).^37^,^38^

1 *r* = *kP*_N_2__^*n*^*P*_H_2__^*h*^*P*_NH_3__^*a*^

As shown in Fig. S1,† H_2_ reaction orders for Cs^+^/Ru/MgO_500red and Ru/La_0.5_Ce_0.5_O_1.75__650red were estimated to be –0.76 and 0.15, respectively. These results indicate that the surface of Cs^+^/Ru/MgO_500red is strongly poisoned by hydrogen. In contrast, Ru/La_0.5_Ce_0.5_O_1.75__650red is not poisoned by hydrogen. These results are in good agreement with the observations shown in Fig. 1d. Furthermore, the N_2_ reaction order for Cs^+^/Ru/MgO_500red was 1.07, which is in accordance with earlier work.^9^,^37^,^39^ In contrast, it was 0.76 for Ru/La_0.5_Ce_0.5_O_1.75__650red, indicating that N≡N bond cleavage, which is the rate-determining step for ammonia synthesis, is relatively promoted over Ru/La_0.5_Ce_0.5_O_1.75__650red. Moreover, stability of Ru/La_0.5_Ce_0.5_O_1.75__650red at 350 °C under 3.0 MPa was examined. When an inline gas purifier was installed for cleaning the H_2_/N_2_ mixture (Fig. S2†), the ammonia-synthesis rate was stable for 50 h, indicating that Ru/La_0.5_Ce_0.5_O_1.75_ shows long-term stability.

### Direct observation of Ru/La_0.5_Ce_0.5_O_1.75__650red without exposure to air

**Fig. 2:**
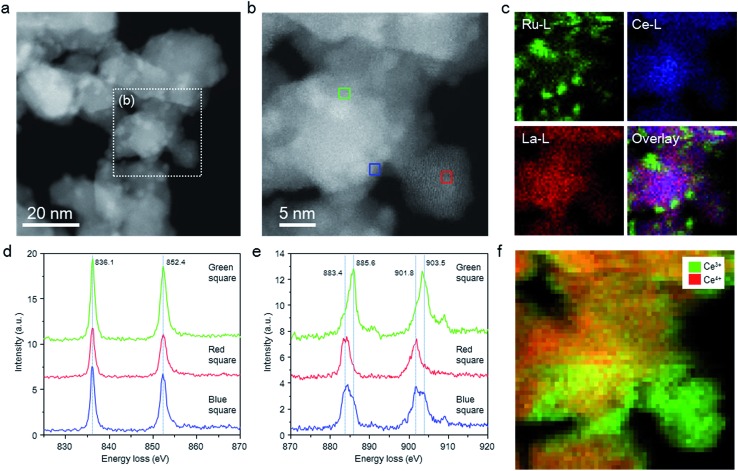
Low-magnification HAADF-STEM images, EDX maps, and EEL spectra of the Ru/La_0.5_Ce_0.5_O_1.75__650red catalyst without exposure to air. (a) and (b) HAADF-STEM images and (c) EDX maps of Ru/La_0.5_Ce_0.5_O_1.75__650red; (d) and (e) EEL spectra of La M_4,5_ (d) and Ce M_4,5_ (e) edges for the areas indicated by the green, blue, and red squares in (b); (f) EELS map of Ce^3+^ and Ce^4+^ for the area indicated by (b).

The structure of the Ru/La_0.5_Ce_0.5_O_1.75__650red catalyst was investigated by means of aberration-corrected transmission electron microscopy (TEM), and the elemental distributions and valence states of the Ce ions were evaluated by means of STEM spectrum imaging of simultaneous energy dispersive X-ray (EDX) mapping and EELS. Because the elemental states and the structure of the catalyst might be changed by exposure to air, we carried out these analyses in the absence of air using a special holder with a gas cell to transfer the sample from an inert gas environment to the inside of the TEM column. Comparison of the high-angle annular dark-field (HAADF) STEM images (Fig. 2a and b) and EDX maps (Fig. 2c) of the catalyst indicated that Ce and La were homogeneously dispersed in the oxide support and that Ru particles were loaded on the support.

Fig. 2d and e shows EEL spectra extracted from the spectrum imaging data for the centre region (Fig. 2b, green square) of a thick catalyst particle (information about both the surface and the bulk of the particle), the edge (blue square) of the same catalyst particle (information mainly about the particle surface), and the centre (red square) of a thin catalyst particle (information about the particle surface). In all of the EEL spectra, two La M_4,5_ peaks assignable to La^3+^ were observed, one at 836.1 and the other at 852.4 eV.^40^ In addition, all of the EEL spectra showed Ce M_4,5_ peaks ascribed to Ce^3+^ and Ce^4+^ at around 883.4 (as split peaks when the intensity was strong) and 901.8 eV and at 885.6 and 903.5 eV, respectively.^40^–^42^ Ce^4+^ predominated in the centre region (green square) of the thick catalyst particle, whereas Ce^3+^ predominated at the edge (blue square) of the thick catalyst particle, and the proportion of Ce^3+^ was highest at the centre (red square) of the thin catalyst particle. EELS maps of Ce in the thick and thin particles clearly showed the same tendency; that is, Ce^3+^ was enriched near the surface of the catalyst particles (Fig. 2f). These results indicate that a substantial proportion of the Ce^4+^ atoms located near the surface of the catalyst particles were reduced to Ce^3+^ at 650 °C.

**Fig. 3:**
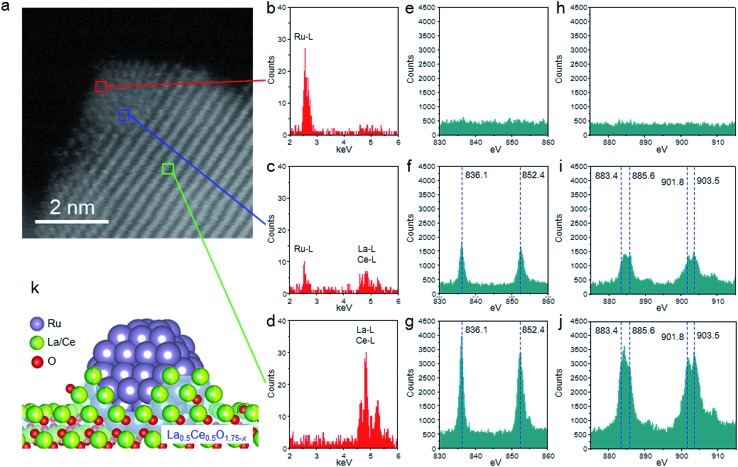
High-magnification HAADF-STEM images, EDX spectra, and EEL spectra of the Ru/La_0.5_Ce_0.5_O_1.75__650red catalyst without exposure to air. (a) HAADF-STEM image. (b)–(d) EDX spectra of areas indicated by the red, blue, and green squares in (a). (e)–(j) EEL spectra of La M_4,5_ (e)–(g) and Ce M_4,5_ (h)–(j) edges for areas indicated by the red, blue, and green squares in (a). (k) Schematic representation of the structure of Ru/La_0.5_Ce_0.5_O_1.75__650red.

We used HAADF-STEM imaging and simultaneous EDX and EELS measurements at a higher magnification to study the interaction between the fine Ru particles and the support (Fig. 3). In the HAADF-STEM images shown in Fig. 3a (see Fig. S3† for additional images of the catalyst), we observed fine Ru particles (diameter ≈ 2 nm) dispersed on the composite-oxide support. Spot EDX and EEL spectra were measured for the detection of Ru and the rare earth elements (La and Ce) and the valence state of the rare earth elements, respectively. In the area indicated by the red square in Fig. 3a, the only observable peak, occurring in the EDX spectrum, was assignable to Ru (Fig. 3b, e and h). In contrast, the middle part of the Ru particle (blue square) showed a Ru peak in the EDX spectrum and peaks for La^3+^, Ce^3+^, and Ce^4+^ in both the EDX and the EEL spectra (Fig. 3c, f and i). The EDX and EEL spectra of the support material (green square) showed only peaks for the constituents of the support, that is, La^3+^, Ce^3+^, and Ce^4+^ (Fig. 3d, g and j). These results revealed that the Ru particles were partially covered by partially reduced support material; this result is consistent with a strong metal-support interaction (SMSI).^26^,^43^,^44^ In addition, these observations clearly indicate that fine Ru particles were anchored to the reduced La_0.5_Ce_0.5_O_1.75_ after pre-reduction at the unusually high temperature of 650 °C (Fig. 3k).

### Explanation of the high ammonia-synthesis ability of Ru/La_0.5_Ce_0.5_O_1.75__650red

We investigated the reason for the high ammonia-synthesis rate over Ru/La_0.5_Ce_0.5_O_1.75__650red. At 1.0 MPa and 350 °C, the ammonia-synthesis rate over Ru/La_0.5_Ce_0.5_O_1.75__500red was approximately 1.7 times the rate over Ru/CeO_2__500red and approximately 2 times the rate over Ru/La_2_O_3__500red (Fig. S4†). These results indicate that use of the La_2_O_3_–CeO_2_ composite support increased the ammonia-synthesis rate. In the X-ray diffraction (XRD) pattern of Ru/La_2_O_3_, many peaks assignable to LaOOH and La(OH)_3_ were observed, in addition to small peaks assigned to La_2_O_3_ (Fig. S5†), compounds that are produced by the adsorption of water vapour from the atmosphere onto La_2_O_3_. Note that adsorption of water decreases the basicity of the support and thus should be avoided. In contrast, the XRD pattern of Ru/La_0.5_Ce_0.5_O_1.75_ was consistent with a cubic fluorite structure like that of CeO_2_, although the peaks were shifted to much lower angles than for the corresponding peaks for Ru/CeO_2_. The XRD pattern contained no peaks assignable to impurities. A plot of the lattice constant as a function of La/(Ce + La) molar ratios for fresh Ru/La_*y*_Ce_1–*y*_O_2–0.5*y*_ (0 ≤ *y* ≤ 0.5) was linear (Fig. S6†), in accordance with Vegard’s law, which indicates that the Ru/La_0.5_Ce_0.5_O_1.75_ catalyst was a solid solution of La species homogeneously dissolved in a cubic fluorite structure. Note also that the peaks for Ru/La_0.5_Ce_0.5_O_1.75_ were broader and less sharp than those for Ru/CeO_2_. These results indicate that formation of the composite oxide interfered with water adsorption by La_2_O_3_ and with the crystal growth of the oxidic support. CeO_2_ reportedly tends not to form hydroxide or carbonate, owing to the symmetric octahedrally coordinated O^2–^ ions surrounding the Ce^4+^ in the cubic fluorite structure, whereas La_2_O_3_ does tend to form hydroxide or carbonate, owing to the asymmetric heptahedral coordination.^45^ It is likely that the water adsorption observed over Ru/La_2_O_3_ was inhibited by incorporation of La^3+^ into the cubic fluorite structure. Furthermore, the specific surface area of Ru/La_0.5_Ce_0.5_O_1.75__500red (Table 1) was much higher than the surface areas of Ru/CeO_2__500red and Ru/La_2_O_3__500red (Table S2†). The co-existence of Ce^4+^ and La^3+^ cations on the oxide surface probably prevented sintering of the oxide.^46^ Because of this enhancement of the stability of the material, the Ru particles that formed on La_0.5_Ce_0.5_O_1.75_ (mean diameter = 1.8 nm) after pre-reduction at 500 °C were finer than those that formed on La_2_O_3_ (mean diameter = 7.8 nm) and on CeO_2_ (mean diameter = 2.4 nm) (see Tables 1 and S2† and the TEM images in Fig. S8 and S9†). In addition, the H/Ru ratio, a measure of Ru dispersion, for Ru/La_0.5_Ce_0.5_O_1.75__500red (Table 1) was 3.5 and 1.7 times the ratios for Ru/La_2_O_3__500red and Ru/CeO_2__500red, respectively (Table S2†). These results revealed that the use of the La_2_O_3_–CeO_2_ composite support increased the number of Ru active sites and thus increased the ammonia-synthesis rate over Ru/La_0.5_Ce_0.5_O_1.75__500red relative to the rates over Ru/La_2_O_3__500red and Ru/CeO_2__500red.

**Table 1:** The physicochemical properties and catalytic performance of Ru/Ce_0.5_La_0.5_O_1.75_ reduced at various temperatures

Reduction temperature (°C)	Specific surface area (m^2^ g^–1^)	H/Ru^*a*^ (—)	Degree of Ce^4+^ reduction^*b*^ (%)	Mean Ru particle size^*c*^ (nm)	TOF^*d*^ (s^–1^)	NH_3_-synthesis rate at 350 °C and 1.0 MPa (mmol g^–1^ h^–1^)
500	47	0.46	23	1.8	0.027	22.1
650	42	0.35	43	1.7	0.051	31.3
800	21	0.11	63	2.7	0.108	20.6

^*a*^Estimated from the H_2_ chemisorption capacity.

^*b*^Calculated from the O_2_ absorption capacity shown in Fig. S7 for the reduced catalysts.

^*c*^Estimated from the STEM images in Fig. S8.

^*d*^TOF, turnover frequency. Calculated from the H/Ru value and the ammonia-synthesis rate.

We also investigated the influence of the catalyst pre-reduction temperature on the ammonia-synthesis rate and on the properties of Ru/La_0.5_Ce_0.5_O_1.75_ (Fig. 1a and Table 1). Increasing the pre-reduction temperature from 500 to 650 °C had little effect on the mean Ru particle diameter (see Fig. S8† for TEM and EDX mapping images of Ru/La_0.5_Ce_0.5_O_1.75_ after pre-reduction at the various temperatures; note that although the TEM image of Ru/La_0.5_Ce_0.5_O_1.75__650red in Fig. S8† was obtained after exposure to air, the mean Ru particle diameter was similar to that measured in the absence of air [Fig. 2]). However, increasing the reduction temperature from 650 to 800 °C increased the mean diameter of the Ru particles to 2.7 nm (owing to sintering of the La_0.5_Ce_0.5_O_1.75_ support) and decreased the specific surface area of the catalyst from 42 to 21 m^2^ g^–1^. On the other hand, the H/Ru ratio decreased gradually as the pre-reduction temperature was increased from 500 to 800 °C. Note that when the reduction temperature was increased from 500 to 650 °C, the H/Ru ratio decreased from 0.46 to 0.35, but the mean diameter of the Ru particles remained unchanged. These results indicate that the surface Ru atoms were partially covered with partially reduced support material, at least after reduction at 650 °C, owing to the SMSI phenomenon, which is consistent with the EDX and EEL spectra (Fig. 3). The driving force for the SMSI is considered to be reduction of a support, such as TiO_2–*x*_ and CeO_2–*x*_, bearing a coordinately unsaturated metal cation.^26^,^43^,^44^ We estimated the degree of Ce^4+^ reduction to Ce^3+^ by measuring the O_2_ absorption capacity of the reduced Ru/La_0.5_Ce_0.5_O_1.75_; the degrees of reduction were determined to be 23% and 43% after pre-reduction at 500 and 650 °C, respectively, revealing that SMSI occurred, especially at the higher temperature. The degree of Ce^4+^ reduction for Ru/La_0.5_Ce_0.5_O_1.75__650red indicates that the average composition of the reduced support was Ce_0.5_La_0.5_O_1.64_. We also observed that the lattice was expanded by pre-reduction, owing both to the formation of Ce^3+^, which has a larger ionic radius than Ce^4+^ (1.14 Å *versus* 0.97 Å in eight coordination), and to the formation of oxygen vacancies. Specifically, the lattice parameter of the cubic fluorite structure of La_0.5_Ce_0.5_O_1.75_, as measured by *in situ* XRD analysis, increased from 0.5577 nm at room temperature to 0.5596 and 0.5603 nm after treatment with H_2_ at 500 and 650 °C, respectively (the XRD patterns are compared in Fig. S10†). Note that we confirmed that the lattice expansion that occurred upon treatment with H_2_ was larger than the thermal expansion observed upon simple heat treatment in air (Fig. S10†). Furthermore, both the SMSI effect and sintering of the Ru particles were greater after reduction at 800 °C than after reduction at the lower temperatures, which we attributed to the drastic decrease in the H/Ru ratio (to 0.11) and to the increase both in the degree of Ce^4+^ reduction (to 63%) and in the mean diameter of the Ru particles (to 2.7 nm) (Table 1).

**Fig. 4:**
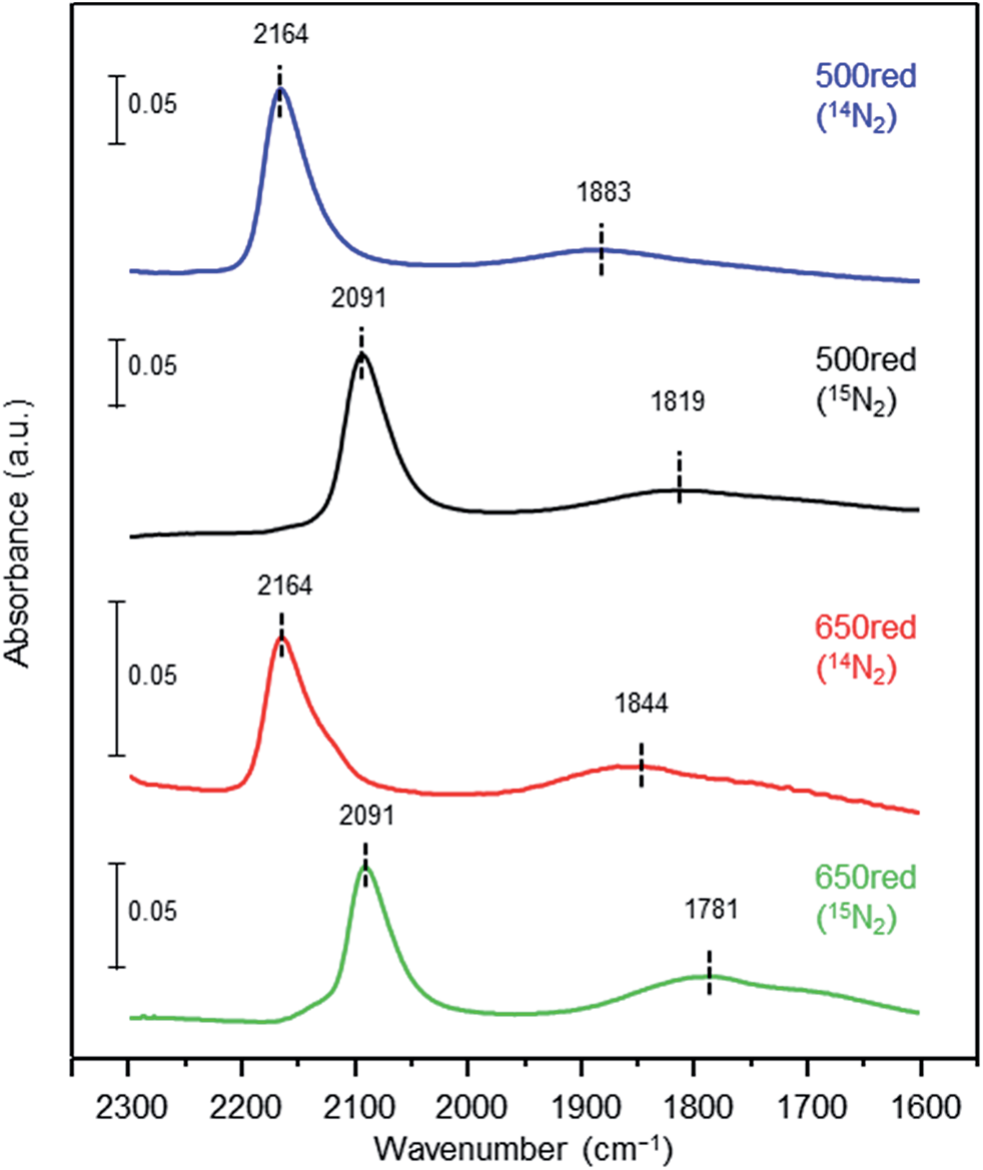
Fourier transform IR spectra of N_2_. Difference infrared spectra of N_2_ (^14^N_2_ and ^15^N_2_) before and after adsorption on Ru/La_0.5_Ce_0.5_O_1.75__500red and Ru/La_0.5_Ce_0.5_O_1.75__650red. The spectra were measured under 6 kPa of N_2_ at 25 °C.

**Fig. 5:**
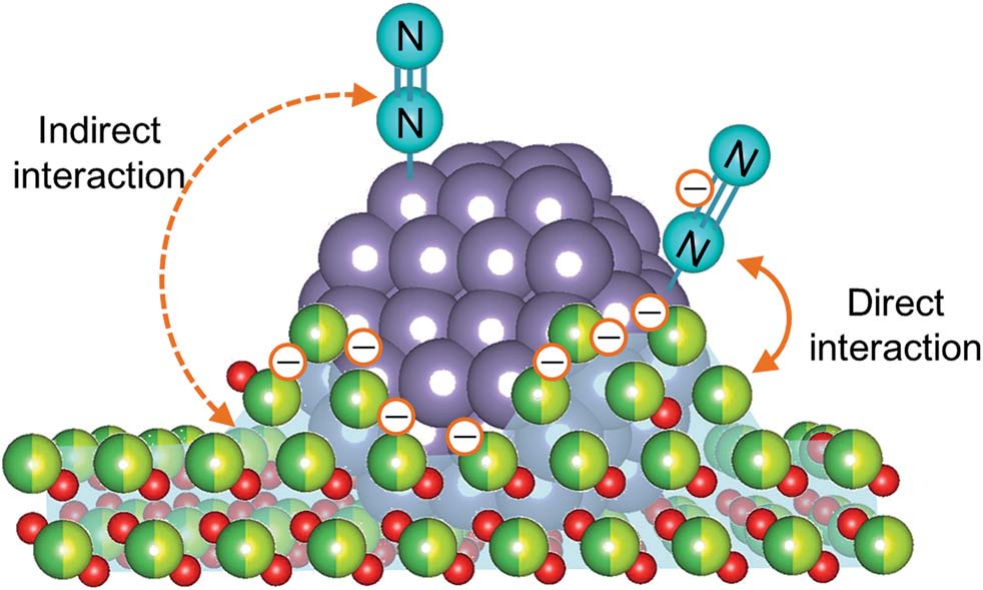
The possible mechanism of N_2_ activation over Ru/Ce_0.5_La_0.5_O_1.75__650red.

To elucidate the influence of the pre-reduction temperature on N≡N bond cleavage, which is the rate-determining step for ammonia synthesis over Ru/La_0.5_Ce_0.5_O_1.75_, we determined the state of the adsorbed N_2_ molecules by means of Fourier transform infrared (IR) spectroscopy. The IR spectra measured after addition of ^14^N_2_ or ^15^N_2_ to Ru/La_0.5_Ce_0.5_O_1.75__500red and Ru/La_0.5_Ce_0.5_O_1.75__650red at room temperature are shown in Fig. 4 (the highest temperature at which our IR cell could be used was 650 °C). Both spectra show a peak at 2164 cm^–1^ and a broader peak at around 1700–1900 cm^–1^. Note that the wavenumber of the broader peak decreased from 1883 to 1844 cm^–1^ when the pre-reduction temperature was increased from 500 to 650 °C. In the spectra measured after ^15^N_2_ adsorption, the two peaks were observed at lower wavenumbers (2091 and 1819 cm^–1^) relative to those for the ^14^N_2_ spectra, and the wavenumbers were in good agreement with those estimated by consideration of the isotope effect:^20^,^47^ 2164 cm^–1^ × (14/15)^1/2^ = 2091 cm^–1^ and 1883 cm^–1^ × (14/15)^1/2^ = 1819 cm^–1^. Similar peak shifts ascribable to the isotope effect were observed in the spectrum after adsorption of ^15^N_2_ on Ru/La_0.5_Ce_0.5_O_1.75__650red. Therefore, all of the peaks were assignable to the stretching vibration mode of N_2_ adsorbed in an end-on orientation on the Ru particles. The peak at 2164 cm^–1^, the location of which was independent of reduction temperature, was assigned to N_2_ adsorbed on Ru atoms that interacted only weakly with the reduced support (Fig. 5, indirect interaction). The broader peaks at around 1700–1900 cm^–1^ were assigned to N_2_ adsorbed on Ru atoms that interacted directly with the reduced support formed by SMSI (Fig. 5, direct interaction). The peak broadening may reflect the heterogeneous character of the metal-support boundary.

Our results indicate that the N≡N bond of N_2_ was weakened by the contribution of SMSI even after reduction at 500 °C, and when the reduction temperature was increased to 650 °C, the contribution of SMSI was greater. That is, the partially reduced support, which is enriched in electrons owing to the reduction of Ce^4+^ to Ce^3+^ and to the formation of oxygen vacancies, partially covered the Ru particles. As a result, electron transfer from the reduced support to the Ru metal was greatly enhanced, and the electrons were transferred to the antibonding π-orbitals of N_2_; thus, the N≡N bonds of N_2_ adsorbed on Ru atoms that interacted directly with the reduced support were further weakened. The ratio of the peak area of the higher-wavenumber peak to that of the lower-wavenumber peak decreased when the pre-reduction temperature was increased from 500 to 650 °C, which is consistent with an increase in the contribution of the SMSI.

These results demonstrate that pre-reduction at high temperature induced SMSI and enhanced the turnover frequency (TOF) but decreased the number of Ru active sites because the Ru particles became partially covered by partially reduced support. The fact that active Ru sites (TOF = 0.051 s^–1^) were abundant (H/Ru = 0.35) after pre-reduction at 650 °C explains the high ammonia-synthesis rate (31.3 mmol g^–1^ h^–1^) over Ru/La_0.5_Ce_0.5_O_1.75__650red. In contrast, after pre-reduction at 800 °C, the Ru sites were very active (TOF = 0.108 s^–1^), but the number of active Ru sites was small (H/Ru = 0.11); thus the ammonia-synthesis rate over Ru/La_0.5_Ce_0.5_O_1.75__800red (20.6 mmol g^–1^ h^–1^) was lower than that over Ru/La_0.5_Ce_0.5_O_1.75__650red. Note that the specific surface area of Ru/CeO_2__650red was only 20 m^2^ g^–1^, the mean diameter of the Ru particles was 3.1 nm, and H/Ru was 0.17, which indicates that sintering of the Ru particles and La_0.5_Ce_0.5_O_1.75_ was retarded in the case of Ru/La_0.5_Ce_0.5_O_1.75__650red, and thus the H/Ru ratio for this catalyst remained high.

## Conclusions

Pre-reduction of conventional supported-metal catalysts is crucial for their activation, because active metal sites are formed on the surface by reduction of metal oxides, and because adsorbates (such as H_2_O and CO_2_) on the surface of the fresh catalyst are removed. However, pre-reduction at an excessively high temperature results in sintering, which decreases the number of active sites. Here, we found that 400–450 °C was usually sufficient to reduce Ru^3+^. However, pre-reduction of Ru/La_0.5_Ce_0.5_O_1.75_ at the unusually high temperature of 650 °C produced a catalyst that showed a high ammonia-synthesis rate under mild reaction conditions (300–400 °C, 0.1–3.0 MPa). This catalyst consisted of fine Ru particles anchored on a heat-tolerant complex-oxidic support. During pre-reduction, the particle size of the Ru particles remained unchanged, but the particles became partially covered with partially reduced La_0.5_Ce_0.5_O_1.75_. A strong interaction between the Ru active sites and the reduced support accelerated the rate-determining step of ammonia synthesis, that is, N≡N bond cleavage. We suggest that this simple strategy for the design of Ru catalysts—that is, using a thermostable composite oxide containing a redox-active rare earth element in a cubic fluorite structure as a support, and pre-reducing the supported catalyst at high temperature—will lead to the development of a more energy efficient ammonia-synthesis process, thus reducing global energy consumption and facilitating the eventual use of ammonia as an energy carrier.

## Conflicts of interest

There are no conflicts to declare.

## Supplementary Material

Supplementary information
