# Supplementary material for: Efficient ammonia synthesis over a Ru/La0.5Ce0.5O1.75 catalyst pre-reduced at high temperature
Source: Chem Sci. 2018 Jan 15;9(8):2230–7. doi: 10.1039/c7sc05343f (PMC5897884; doi:10.1039/c7sc05343f)
Supplement: Supplementary file 1 [file SC-009-C7SC05343F-s001.pdf]

Electronic Supplementary Material (ESI) for Chemical Science.
This journal is © The Royal Society of Chemistry 2018

Electronic supplementary information

Efficient ammonia synthesis over Ru/$La_{0.5}Ce_{0.5}O_{1.75}$ pre-reduced at high temperature

Yuta Ogura,[1] Katsutoshi Sato,[1,2] Shin-ichiro Miyahara,[1] Yukiko Kawano,[1] Takaaki Toriyama,[3] Tomokazu Yamamoto,[4] Syo Matsumura,[3,4] Saburo Hosokawa,[2] and Katsutoshi Nagaoka[1]*

[1] *Department of Integrated Science and Technology, Faculty of Science and Technology, Oita University, 700 Dannoharu, Oita 870-1192, Japan*

[2] *Elements Strategy Initiative for Catalysts and Batteries, Kyoto University, 1-30 Goryo-Ohara, Nishikyo-ku, Kyoto 615-8245, Japan*

[3] *The Ultramicroscopy Research Center, Kyushu University, Motooka 744, Nishi-ku, Fukuoka 819-0395, Japan*

[4] *Department of Applied Quantum Physics and Nuclear Engineering, Kyushu University, Motooka 744, Nishi-ku, Fukuoka 819-0395, Japan*

1. Details of experimental methods

Catalyst preparation

The $La_{0.5}Ce_{0.5}O_{1.75}$ support was prepared at room temperature by means of a co-precipitation method from a suspension formed by the addition of a solution containing $La(NO_3)_3 \cdot 6H_2O$ (Wako Pure Chemical, Japan) and $Ce(NO_3)_3 \cdot 6H_2O$ (Kanto Chemical, Japan) to a 28 wt % ammonia solution (Wako). The precipitate was held in the suspension for at least 1 h with stirring and was then filtered, washed with distilled water, dried at 80 °C for at least a half day, and calcined at 700 °C in static air for 5 h. The CeO_2, La_2O_3, Pr_6O_{11}, and MgO supports were prepared by precipitation at room temperature from the corresponding nitrates in a manner similar to that described for the $La_{0.5}Ce_{0.5}O_{1.75}$ support. $Pr(NO_3)_3 \cdot 6H_2O$ (Kanto Chemical) and $Mg(NO_3)_2 \cdot 6H_2O$ (Wako) were used as precursors for Pr_6O_{11} and MgO, respectively.

All of the supports were impregnated with $Ru_3(CO)_{12}$ (Furuya Metal, Japan) in tetrahydrofuran (THF, Wako). The Ru loading was fixed at 5 wt % for each catalyst. A THF suspension containing $Ru_3(CO)_{12}$ and the support was stirred for 12 h, and then the solvent was removed by rotary evaporation. The obtained powder was kept at 80 °C for 18 h under air and then heated to 500 °C under an Ar stream and kept at that temperature for 5 h to remove the CO ligand from the $Ru_3(CO)_{12}$. Doping of Ru/MgO with Cs_2O was carried out as described previously.[S1] In addition, Ru/MgO was impregnated with Cs_2CO_3 (Wako) in absolute ethanol (Wako) as follows. A suspension of Cs_2CO_3 and MgO in ethanol was stirred for 3 h, and then the solvent was removed by rotary evaporation. The obtained powder was kept under a vacuum for 18 h. The Cs/Ru atomic ratio was fixed at 1.0.

Ammonia-synthesis activity tests

Ammonia-synthesis rates were measured with a conventional flow system under either atmospheric pressure or high pressure, as described previously.[S2] Briefly, powdered catalysts

were pressed into pellets at 20 MPa for 5 min, crushed, and sieved to grains with diameters of 250–500 μm. A tubular Inconel reactor (i.d. = 7 mm) was packed with quartz wool, and then 100 mg of catalyst was added. Research-grade gases (>99.99%) were supplied from high-pressure cylinders without further purification. On the other hand, only for long-term test, inline gas purifier (MicroTorr MC50-904FV, SAES Pure Gas, USA) was used, and then concentration of impurities (H_2O, O_2, CO_2) in gas mixture was reduced to below 100 ppt. The catalysts were reduced in a flow of pure H_2 (60 NmL min^{-1}) at 450, 500, 650, or 800 °C for 1 h at 0.1 MPa and then cooled to 300 °C in an Ar stream. After the pressure was adjusted to 0.1, 0.5, 1.0, or 3.0 MPa at 300 °C, a 3:1 (mol/mol) H_2/N_2 mixture (space velocity = 72,000 NmL h^{-1} g^{-1}) was fed to the catalyst. The temperature of the catalyst was kept constant for 0.5 h to facilitate measurement of ammonia-synthesis rates. The catalyst was then heated to 400 °C in 25 °C increments. The ammonia-synthesis rate was determined from the rate of decrease of electron conductivity (CM-30R, DKK-TOA, Japan) of a dilute sulfuric acid solution (1–100 M) used to trap the ammonia produced under the reaction conditions.

Kinetic analysis

Reaction kinetics were analyzed by a reported method.[S3,S4] In brief, the reaction orders with respect to the N_2, H_2, and NH_3 were determined by measuring N_2, H_2, and NH_3 pressure dependence for the ammonia-synthesis rates with the assumption of the rate expression (1). Equations (2) to (5) were also used for these analyses.

$$r = kP_{N_2}^{n}P_{H_2}^{h}P_{NH_3}^{a} \quad (1)$$

$$r = \frac{(1/w)dy_o}{d(1/q)} \quad (2)$$

$$\log y_o = \log\left(\frac{C}{q}\right)^{\frac{1}{m}} \quad (3)$$

$$r = \left(\frac{1}{w}\right)\left(\frac{C}{m}\right)y_{\mathrm{o}}^{(1-m)} \qquad (4)$$

$$C = k_2 P_{\mathrm{H_2}}^{h} P_{\mathrm{N_2}}^{n} \qquad (5)$$

r, w, y_0, q, C, and (1-m) denotes ammonia-synthesis rate, catalyst mass, ammonia mole fraction at the reactor outlet, flow rate, constant and a. Kinetic analyses were performed at 350 °C and 0.1 MPa. Other reaction conditions are described in Table S1 and Fig S1. To avoid the contribution of the reverse reaction, kinetic measurements were carried out at a GHSV value where the ammonia concentration at the reactor exit was far away from the thermodynamic equilibrium concentration.

Characterisation of the supported Ru catalysts

High-angle annular dark-field scanning transmission electron microscopy (HAADF-STEM) images and energy dispersive X-ray (EDX) elemental maps were obtained with a JEM-ARM200CF electron microscope (JEOL, Japan) operated at 120 or 200 kV. Electron energy loss spectroscopy was performed at an acceleration voltage of 80 kV to reduce damage to the sample by the electron beam. For STEM and electron energy loss spectroscopy, powdered catalyst samples reduced in a quartz reactor at 650 °C under a H_2 flow were crushed and deposited on TEM grids coated with a thin carbon film in a glove box. The samples thus prepared were transferred from the glove box to the inside of the TEM column without being exposed to the air by means of a special holder with a gas cell. For other measurements, samples were dispersed in ethanol under ambient conditions, and the dispersion was dropped onto a carbon-coated copper grid and then dried under a vacuum at ambient temperature for 24 h.

X-ray diffraction (XRD) analysis was performed with a SmartLab X-ray diffractometer (Rigaku, Japan) equipped with a Cu Kα radiation source. For *in situ* measurements, the sample was

placed in a reactor chamber (XRK 900, Anton Parr, Austria) and heated at 500 or 650 °C for 1 h under a stream of H_2 or a stream of 1:4 (v/v) O_2/N_2, and then the diffraction patterns were observed at 500 or 650 °C under the corresponding gas stream. PDXL2 software (Rigaku) with ICDD, COD,[S5] and AtomWork[S6] databases was used to analyse the XRD patterns

The specific surface areas of the catalysts after N_2 treatment at 300 °C were determined by the Brunauer–Emmett–Teller method using a BELSORP-mini instrument (BEL Japan Inc., Japan).

H_2 chemisorption capacity was measured to estimate the Ru dispersion of the catalysts. H_2 was fed to each sample at 60 NmL min^{-1}, and the temperature was increased from room temperature to 500, 650, or 800 °C. The sample was maintained at the desired temperature for 1 h in the H_2 flow and was then purged with a stream of Ar for 30 min, cooled to −78 °C, and flushed with Ar for 60 min. After this pretreatment, H_2 chemisorption analysis was carried out at −78 °C in an Ar stream (60 NmL min^{-1}) by means of a pulsed-chemisorption technique.

The O_2 absorption capacity of the catalyst, which is a measure of the degree of catalyst reduction, was measured by means of a pulse injection method. The amount of O_2 absorbed on the reduced catalyst was sequentially measured at 25, 450, and 800 °C as follows. A catalyst sample (200 mg) was reduced *in situ* at 500, 650, or 800 °C for 1 h, kept in Ar at corresponding temperature for 30 min and then cooled to room temperature under Ar. At the temperature, pulses of pure O_2 (124 μmol/pulse) were injected into the catalyst bed until the level of O_2 detected by a thermal conductivity detector stabilised, an indication that the maximum amount of O_2 had been adsorbed on the catalyst. After the O_2 absorption measurement, the catalyst was heated to 450 °C in an Ar flow, and O_2 absorption at 450 °C was again measured by means of the above-described method. In addition, each catalyst was heated to 800 °C in an Ar flow, and

O_2 absorption at 800 °C was measured by the same method.

Infrared spectra of adsorbed N_2 were measured with a spectrometer (FT/IR-6600, JASCO, Japan) equipped with a mercury–cadmium–tellurium detector at a resolution of 4 cm^{-1} as described previously.[S2] Samples (~20 mg) were pressed into self-supporting disks (10 mm in diameter). Each disk was placed in a silica-glass cell equipped with CaF_2 windows and was then connected to a closed gas-circulation system. The disk was pretreated with circulated H_2 (80 kPa) that had been passed through a liquid N_2 trap. The disk was heated from room temperature to 500 or 650 °C and then kept at that temperature for 1 h. After the reduction, the cell containing the disk was evacuated at the same temperature for 0.5 h to remove the H_2. After this pretreatment, the disk was cooled to room temperature under a vacuum. Pure N_2 (>99.9995%) was supplied to the system via a liquid N_2 trap. $^{15}N_2$ (98%) was used without purification. The infrared spectrum of the sample at room temperature before N_2 adsorption was used as the background, and difference spectra were obtained by subtracting the background spectra from the spectra of the samples containing adsorbed N_2.

2. Supporting Results

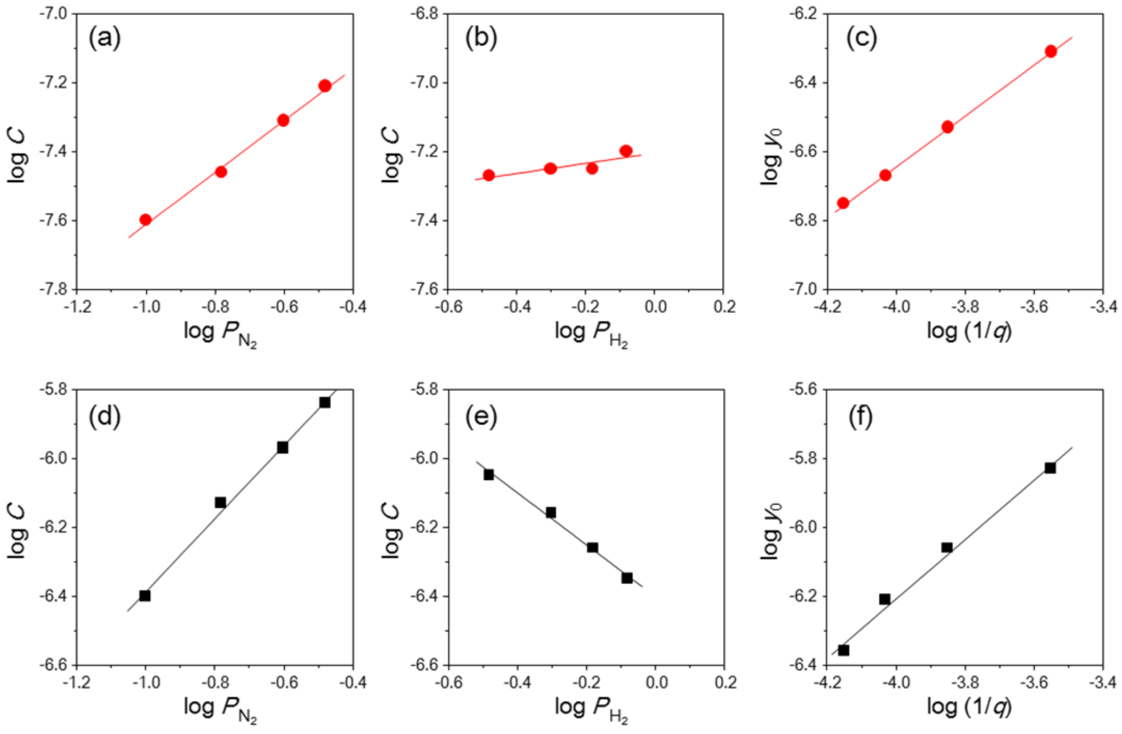

Fig. S1. Graphs for estimating reaction orders over Ru/$La_{0.5}Ce_{0.5}O_{1.75}$_650red and Cs^+/Ru/MgO_500red at 350 °C and 0.1 MPa. (a) and (d) N_2. (b) and (e) H_2. (c) and (f) NH_3. Figs (a)-(c) are for Ru/$La_{0.5}Ce_{0.5}O_{1.75}$_650red; and figs (d)-(f) are for Cs^+/Ru/MgO_500red.

Table S1. Reaction conditions and ammonia-synthesis rates for kinetic analyses over $Ru/La_{0.5}Ce_{0.5}O_{1.75}$_650red and Cs^+/Ru/MgO_500red

$Ru/La_{0.5}Ce_{0.5}O_{1.75}$_650red

Weight (mg)		Flow rate (NmL min^{-1})			GHSV (NL h^{-1}g^{-1})	NH_3 synthesis rate (mmol h^{-1} g^{-1})	Yield/Yield_Eq. (-)	Orders
		N_2	H_2	Ar				
5.0	for N_2	24	120	96	2,880	18.75	0.04	
		40	120	80	2,880	23.79	0.03	0.76
		60	120	60	2,880	30.64	0.04	(*n*)
		80	120	40	2,880	36.41	0.05	
	for H_2	40	80	120	2,880	32.16	0.06	
		40	120	80	2,880	33.22	0.04	0.15
		40	160	40	2,880	33.22	0.04	(*h*)
		40	200	0	2,880	36.05	0.05	
	for NH_3	20	60	0	960	20.91	0.05	
		40	120	0	1,920	25.24	0.03	−0.36
		60	180	0	2,880	27.76	0.02	(*a*)
		80	240	0	3,840	30.28	0.02	

Cs^+/Ru/MgO_500red

Weight (mg)		Flow rate (NmL min^{-1})			GHSV (NL h^{-1}g^{-1})	NH_3 synthesis rate (mmol h^{-1} g^{-1})	Yield/Yield_Eq. (-)	Orders
		N_2	H_2	Ar				
50.0	for N_2	24	120	96	288	4.09	0.09	
		40	120	80	288	7.07	0.09	1.07
		60	120	60	288	9.67	0.12	(*n*)
		80	120	40	288	12.64	0.16	
	for H_2	40	80	120	288	8.24	0.16	
		40	120	80	288	6.67	0.09	−0.76
		40	160	40	288	5.38	0.07	(*h*)
		40	200	0	288	4.52	0.06	
	for NH_3	20	60	0	96	6.32	0.16	
		40	120	0	192	7.44	0.10	−0.15
		60	180	0	288	7.81	0.07	(*a*)
		80	240	0	384	7.44	0.05	

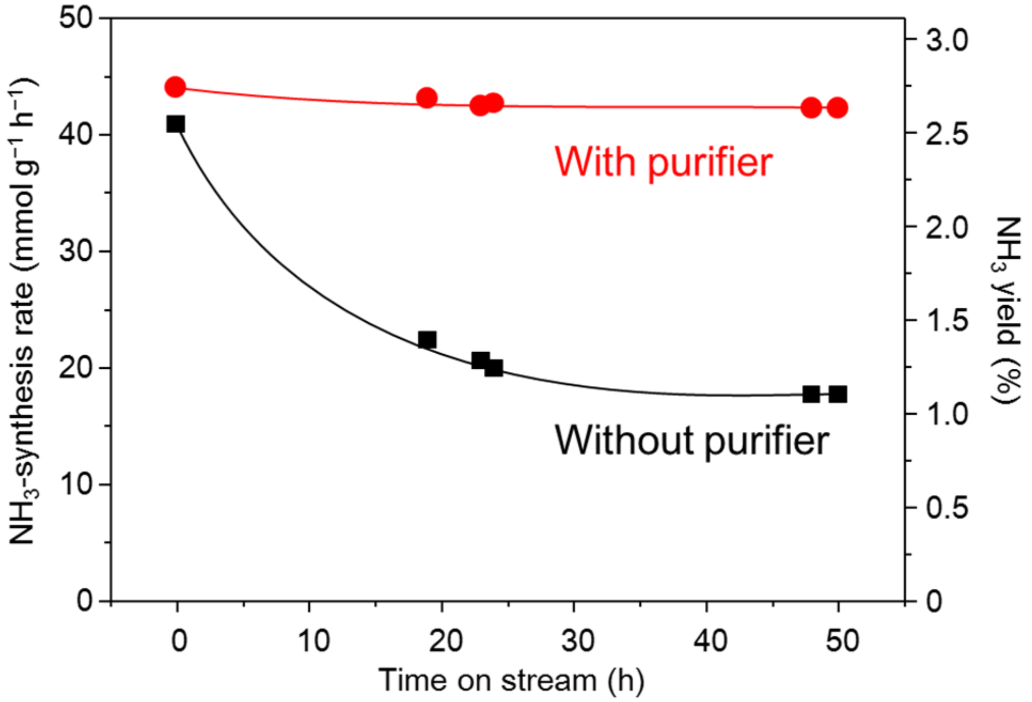

Fig. S2. Time course of ammonia formation rate over Ru/$La_{0.5}Ce_{0.5}O_{1.75}$_650red with and without using gas purifier. Reaction conditions: catalyst, 100 mg; synthesis gas, H_2/N_2 = 3 with a flow rate of 120 NmL min^{-1}; pressure, 3.0 MPa; reaction temperature, 350 °C. When inline gas purifier is used, concentration of impurities (H_2O, O_2, CO_2) in gas mixture is reduced to below 100 ppt.

Ammonia-synthesis rate of Ru/$La_{0.5}Ce_{0.5}O_{1.75}$_650red was stable for 50 h when inline gas purifier was used. On the other hand, the rate decreased gradually without using gas purifier, indicating that the impurities in the H_2/N_2 mixture degraded the catalyst.

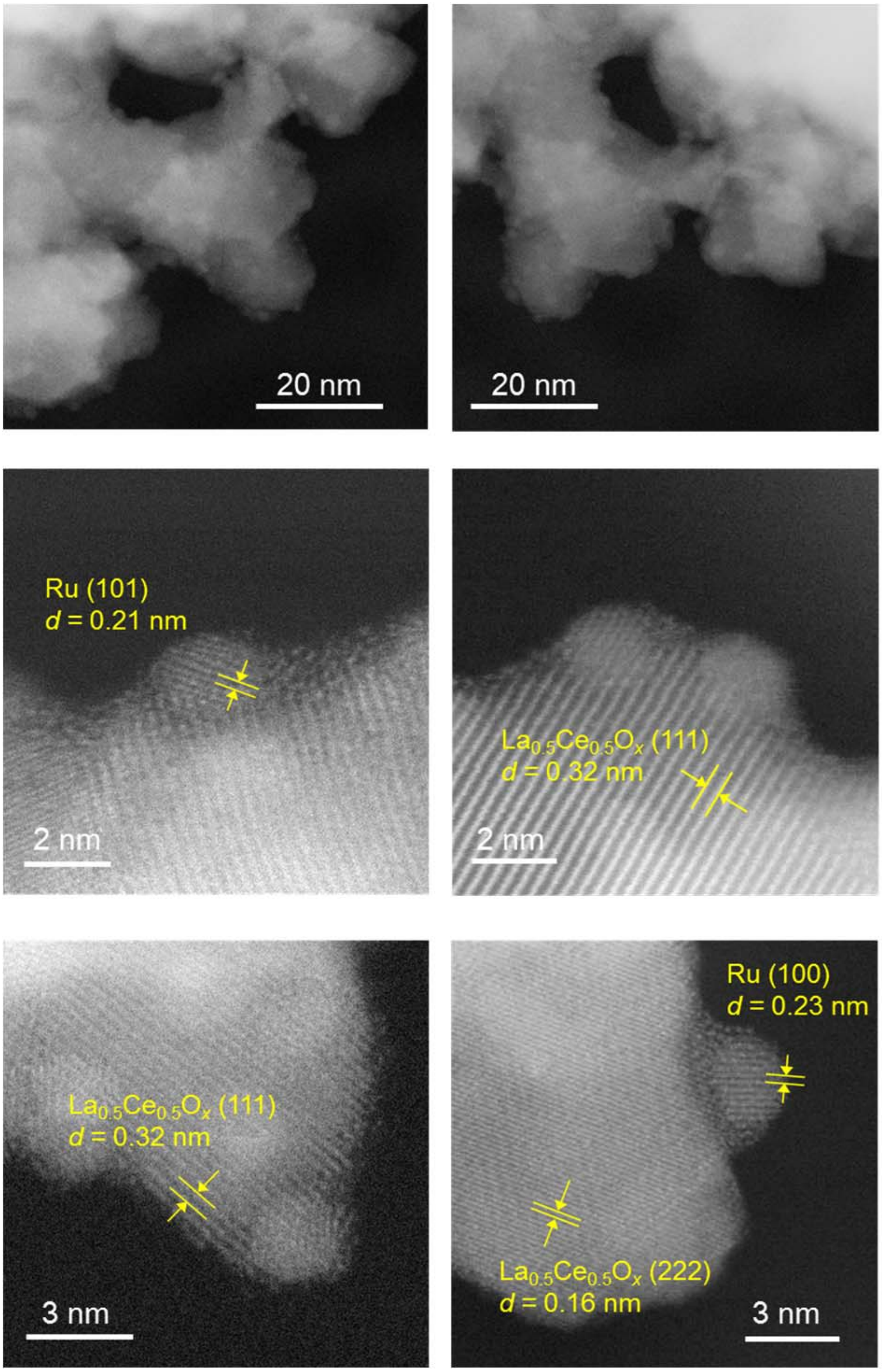

Fig. S3. HAADF-STEM images of Ru/$La_{0.5}Ce_{0.5}O_{1.75}$_650red without exposure to air.

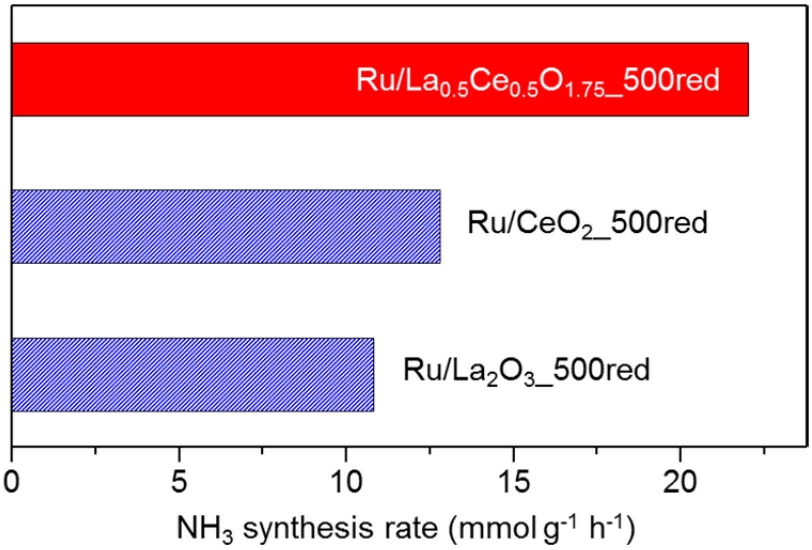

Fig. S4. Ammonia-synthesis rates over Ru/$La_{0.5}Ce_{0.5}O_{1.75}$_500red, Ru/CeO_2_500red, and Ru/La_2O_3_500red. Reaction conditions: catalyst, 100 mg; pre-reduction temperature, 500 °C, reactant gas, 3:1 H_2/N_2 at a flow rate of 120 NmL min^{-1}; pressure, 1.0 MPa; temperature, 350 °C.

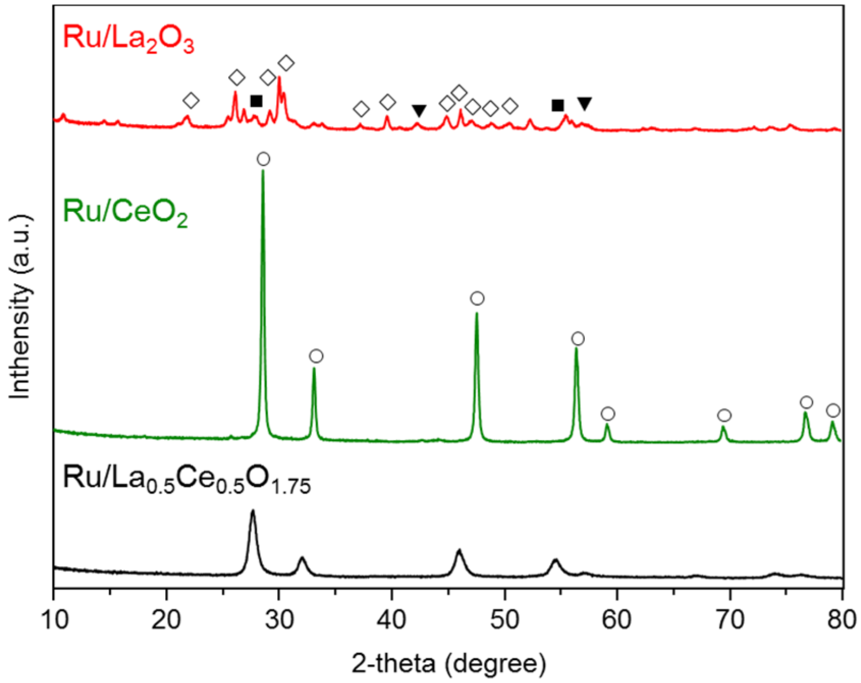

Fig. S5. XRD patterns of fresh Ru/La_2O_3 (red), fresh Ru/CeO_2 (green), and fresh $Ru/La_{0.5}Ce_{0.5}O_{1.75}$ (black). ◊, LaOOH; ■, $La(OH)_3$; ▼ La_2O_3; ○, CeO_2.

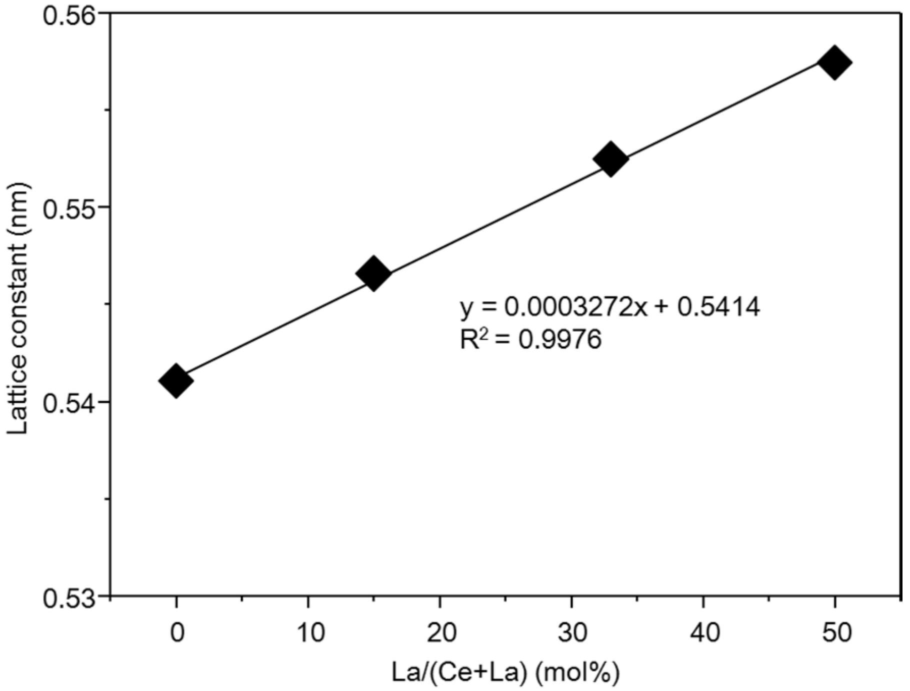

Fig. S6. Lattice constants as a function of La/(Ce + La) molar ratios for fresh Ru/La$_y$Ce$_{1-y}$O$_{2-0.5y}$ ($0 \leq y \leq 0.5$). Lattice constants were calculated by assuming a cubic fluorite structure and using the *d* value of the (311) plane.

Table S2. Physicochemical properties and catalytic performances of supported Ru catalysts

Catalyst	Reduction temperature (°C)	Specific surface area ($m^2\ g^{-1}$)	H/Ru[a] (-)	Mean Ru particle size[b] (nm)	NH_3-synthesis rate at 350 °C and 1.0 MPa ($mmol\ g^{-1}\ h^{-1}$)
Ru/La_2O_3	500	18	0.13	7.8	10.6
Ru/CeO_2	500	24	0.27	2.4	12.8
Ru/CeO_2	650	20	0.17	3.1	17.2

[a] Estimated from the H_2 chemisorption capacity.

[b] Estimated from the STEM images shown in Fig. S9.

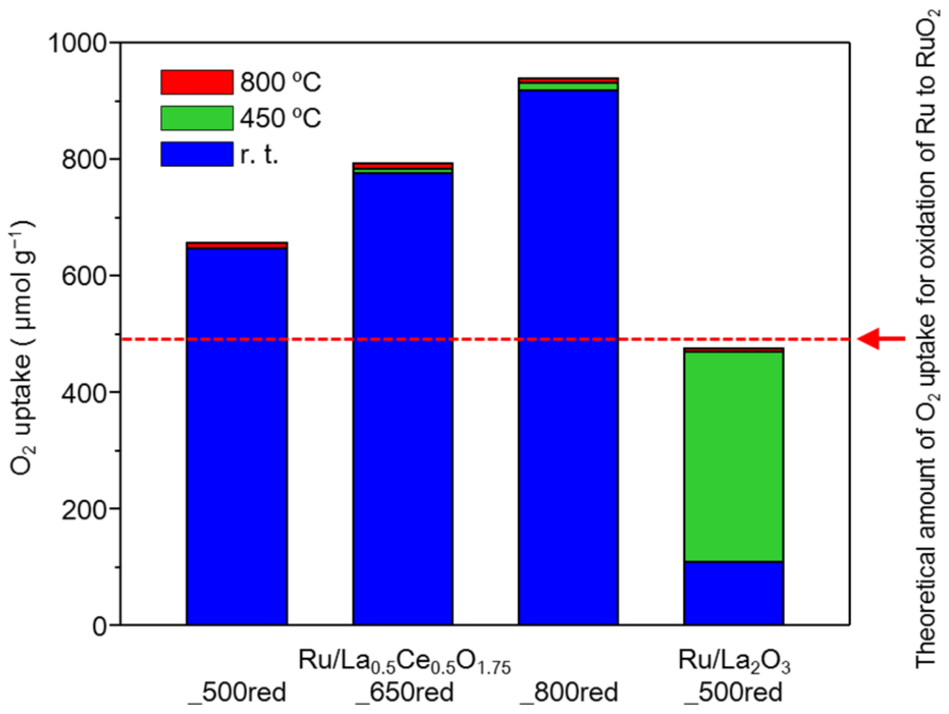

Fig. S7. O_2 absorption capacity of Ru/La_2O_3_500red, Ru/$La_{0.5}Ce_{0.5}O_{1.75}$_500red, Ru/$La_{0.5}Ce_{0.5}O_{1.75}$_650red, and Ru/$La_{0.5}Ce_{0.5}O_{1.75}$_800red. After the catalysts were pre-reduced at the specified temperature, O_2 absorption was measured sequentially at room temperature (r.t.), 450 °C, and 800 °C.

To estimate the degree of reduction of Ce^{4+} to Ce^{3+} in the Ru/$La_{0.5}Ce_{0.5}O_{1.75}$ after pre-reduction, we determined the O_2 absorption capacity of the reduced catalysts by pulsing O_2 into the catalyst bed first at 25 °C and then sequentially at 450 and 800 °C. The O_2 absorption capacities and calculated values for the degree of reduction of Ce^{4+} to Ce^{3+} are shown in Fig. S7 and Table 1, respectively. Note that mass spectroscopic analysis of the exit gas did not reveal any other products (such as H_2O) during the experiments. At room temperature, Ru/La_2O_3_500red adsorbed O_2 at 107 μmol g^{-1}. After the initial O_2 absorption measurement, the

catalyst was heated to 450 °C in Ar and then exposed to another O_2 pulse. During the second exposure, O_2 adsorption was 361 μmolg^{-1}. Finally, the catalyst was heated to 800 °C in Ar and again exposed to O_2 pulses. During this third exposure, only trace amounts of O_2 (4.43 μmol g^{-1}) were taken up. The total O_2 absorption capacity of Ru/La_2O_3_500red was nearly equal to the theoretical amount of O_2 uptake for oxidation of Ru to RuO_2 (495 μmol g^{-1}). These results indicate that only the Ru atoms near the surface of large Ru particles (mean diameter, 7.8 nm) were oxidised at room temperature and that the Ru atoms in the bulk were not oxidised until the temperature reached 450 °C. In contrast, at room temperature, Ru/$La_{0.5}Ce_{0.5}O_{1.75}$_500red took up more O_2 than the amount required for oxidation of Ru. These results indicate that some of the Ce^{4+} in $La_{0.5}Ce_{0.5}O_{1.75}$ was reduced to Ce^{3+} during pre-reduction with H_2 and that oxidation of $La_{0.5}Ce_{0.5}O_{1.75-x}$ increased the amount of O_2 absorption at room temperature. During the O_2 absorption measurements at 450 and 800 °C, only trace amounts of O_2 were taken up. These results demonstrate that Ru and $La_{0.5}Ce_{0.5}O_{1.75-x}$ were nearly fully oxidised to RuO_2 and $La_{0.5}Ce_{0.5}O_{1.75}$ at room temperature and that the resulting material was thermally stable. Total O_2 absorption capacity increased as the pre-reduction temperature of Ru/$La_{0.5}Ce_{0.5}O_{1.75}$ was increased from 500 to 800 °C, an indication that the degree of reduction of Ce^{4+} to Ce^{3+} increased with increasing pre-reduction temperature. Assuming that complete oxidation to $La_{0.5}Ce_{0.5}O_{1.75}$ and RuO_2 occurred, the values of the degree of reduction of Ce^{4+} to Ce^{3+} after pre-reduction at 500, 650, and 800 °C were determined to be 23%, 43%, and 63%, respectively.

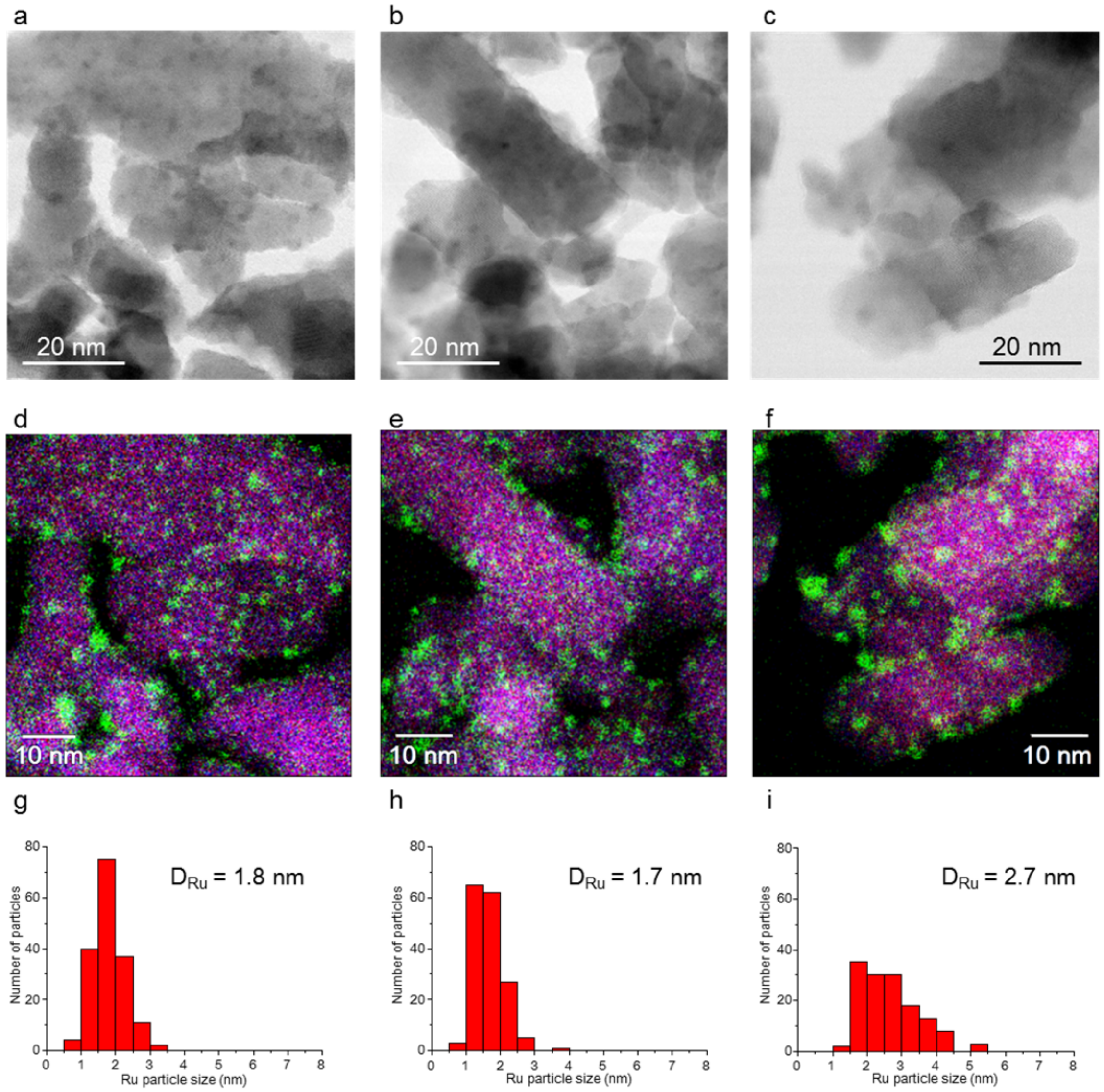

Fig. S8. STEM images and EDX maps for Ru/$La_{0.5}Ce_{0.5}O_{1.75}$_500red, _650red and _800red. (a)–(c) STEM images. (d)–(f) Reconstructed overlay EDX maps of Ru-L (green), La-L (red), and Ce-L (blue). (g)–(i) Ru particle size distributions. Panels (a), (d), and (g) are for Ru/$La_{0.5}Ce_{0.5}O_{1.75}$_500red; panels (b), (e), and (h) are for Ru/$La_{0.5}Ce_{0.5}O_{1.75}$_650red; and panels (c), (f), (i) are for Ru/$La_{0.5}Ce_{0.5}O_{1.75}$_800red.

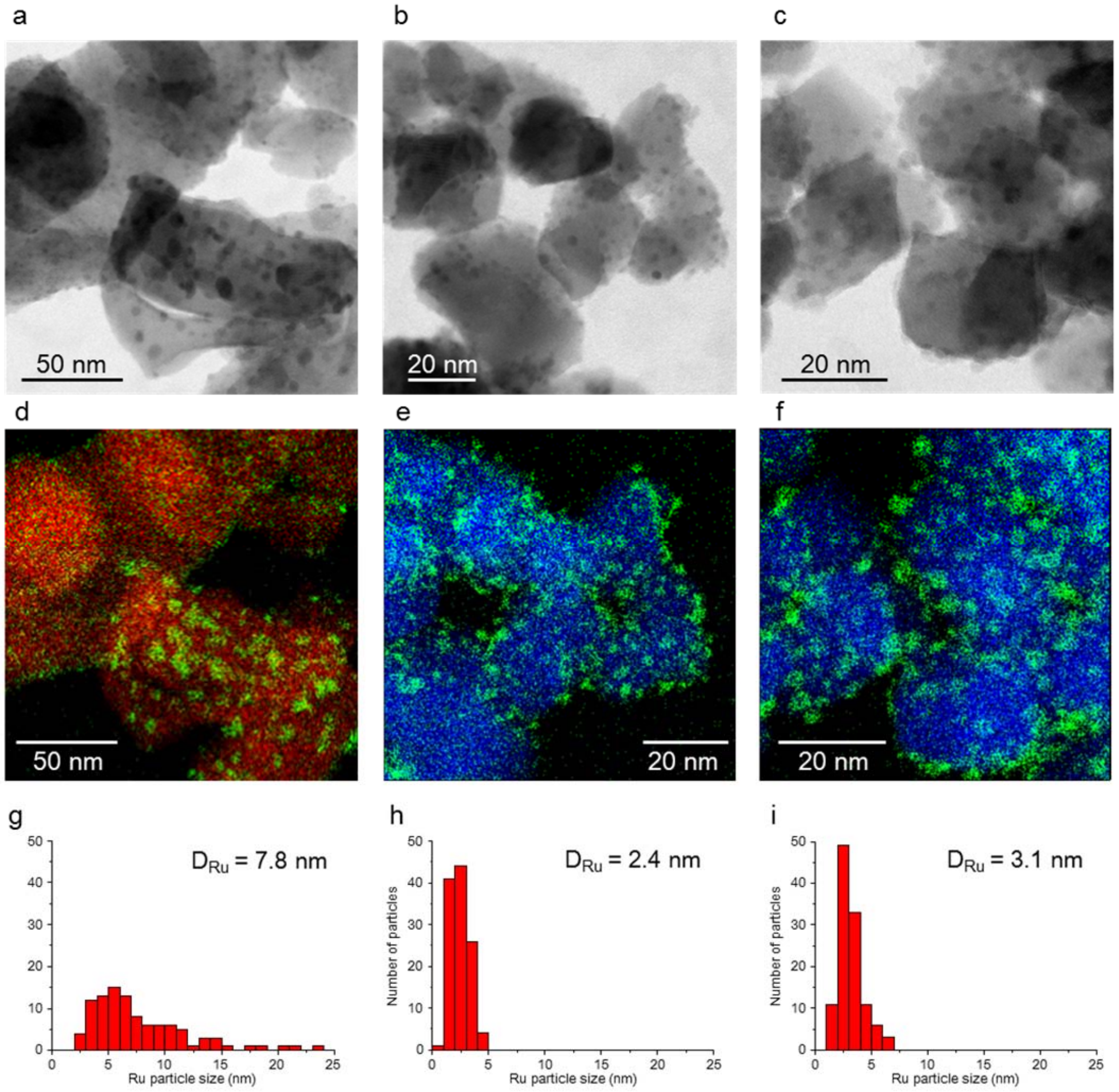

Fig. S9. STEM images and EDX maps of supported Ru catalysts. (a)–(c) STEM images. (d)–(f) Reconstructed overlay EDX maps of Ru-L (green), La-L (red), and Ce-L (blue). (g)–(i) Ru particle size distributions. Panels (a), (d), and (g) are for Ru/La_2O_3_500red; panels (b), (e), and (h) are for Ru/CeO_2_500red; and panels (c), (f), (i) are for Ru/CeO_2_650red.

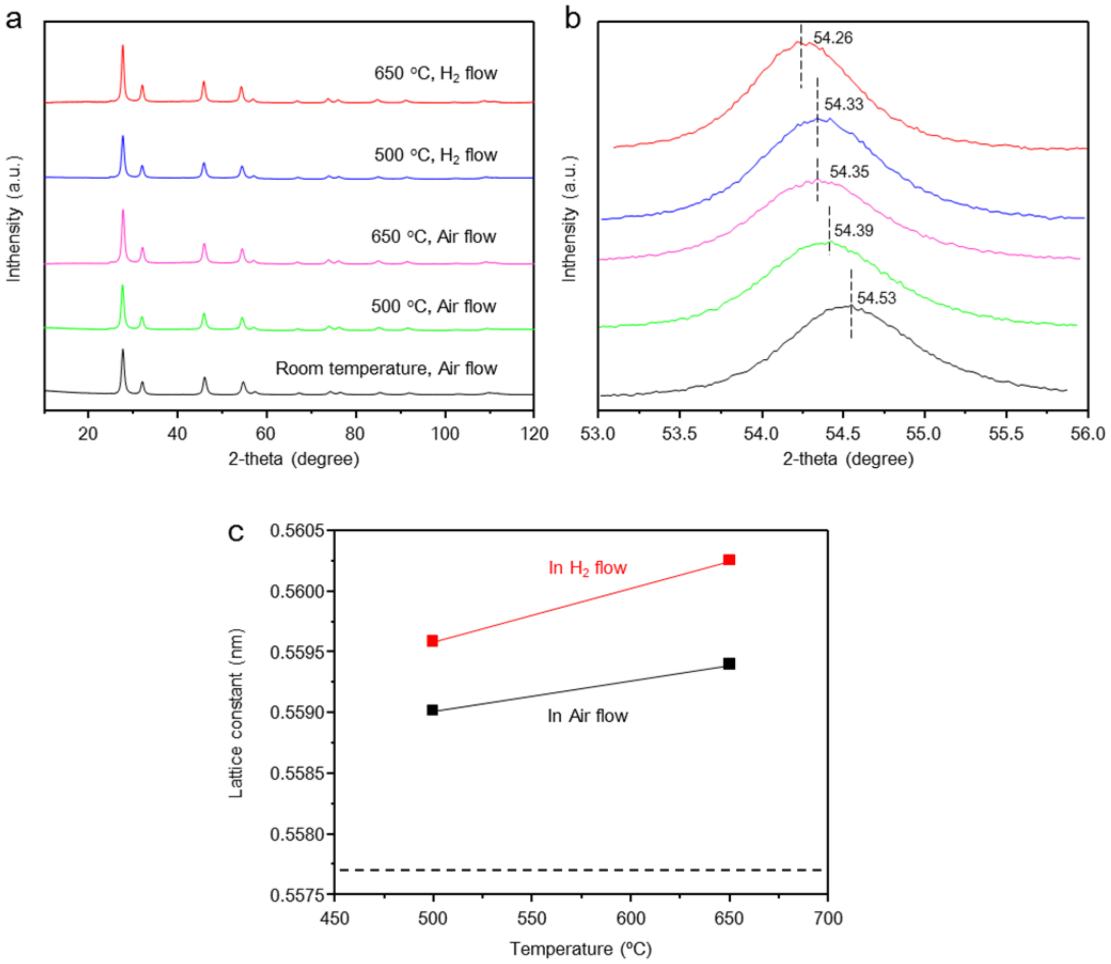

Fig. S10. *In situ* XRD patterns for Ru/$La_{0.5}Ce_{0.5}O_{1.75}$ after various treatments. (a) *In situ* XRD patterns measured after treatment of the catalyst in a flow of H_2 or air at 500 or 650 °C for 1 h (b) Detail showing diffraction peaks of the (311) plane. (c) Temperature dependence of the lattice constant calculated by assuming cubic fluorite structure using the *d* value of the (311) plane. The dotted line indicates the lattice constant of $La_{0.5}Ce_{0.5}O_{1.75}$ measured at room temperature in an air flow.

3. References

[S1] F. Rosowski, A. Hornung, O. Hinrichsen, D. Herein, M. Muhler and G. Ertl, *Appl. Catal., A*, 1997, **151**, 443-460.

[S2] K. Sato, K. Imamura, Y. Kawano, S.-i. Miyahara, T. Yamamoto, S. Matsumura and K. Nagaoka, *Chem. Sci.*, **2017**, 8, 674-679.

[S3] K. Aika, J. Kubota, Y. Kadowaki, Y. Niwa and Y. Izumi, *Appl. Surf. Sci.*, **1997**, 121-122, 488-491.

[S4] R. Kojima and K. Aika, *Appl. Catal., A*, **2001**, 218, 121-128.

[S5] S. Grazulis, D. Chateigner, R. T. Downs, A. F. Yokochi, M. Quiros, L. Lutterotti, E. Manakova, J. Butkus, P. Moeck and A. Le Bail, *J. App.l Crystallogr.*, **2009**, 42, 726-729.

[S6] Y. Xu, M. Yamazaki and P. Villars, *Jpn. J. App. Phys.*, **2011**, 50, 11RH02.

4. Author contributions

K.N. directed and conceived this project. K.S. coordinated the experimental work. O.Y., S. Miyahara, and Y.K. prepared the catalysts, carried out several characterisations, and measured the catalyst activities. T.T., T.Y., and S. Matsumura conducted HAADF-STEM observations and measured EEL and EDX spectra. S.H. conducted the XRD analysis. All the authors discussed the results and commented on the study. O.Y., K.S., and K.N. co-wrote the manuscript.
